# A cross-sectional pilot study of the Scottish early development instrument: a tool for addressing inequality

**DOI:** 10.1186/1471-2458-13-1187

**Published:** 2013-12-17

**Authors:** Lisa Marks Woolfson, Rosemary Geddes, Stephanie McNicol, Josephine N Booth, John Frank

**Affiliations:** 1 School of Psychological Sciences and Health, University of Strathclyde, 40 George St, Glasgow G1 1QE, UK; 2 Scottish Collaboration for Public Health Research and Policy, University of Edinburgh, 20 West Richmond Street, Edinburgh EH8 9DX, UK

**Keywords:** Early development instrument, Child development, Scotland, Socioeconomic factors, Health inequalities/disparities

## Abstract

**Background:**

Early childhood is recognised as a key developmental phase with implications for social, academic, health and wellbeing outcomes in later childhood and indeed throughout the adult lifespan. Community level data on inequalities in early child development are therefore required to establish the impact of government early years’ policies and programmes on children’s strengths and vulnerabilities at local and national level. This would allow local leaders to target tailored interventions according to community needs to improve children’s readiness for the transition to school. The challenge is collecting valid data on sufficient samples of children entering school to derive robust inferences about each local birth cohort’s developmental status. This information needs to be presented in a way that allows community stakeholders to understand the results, expediting the improvement of preschool programming to improve future cohorts’ development in the early years. The aim of the study was to carry out a pilot to test the feasibility and ease of use in Scotland of the 104-item teacher-administered Early Development Instrument, an internationally validated measure of children’s global development at school entry developed in Canada.

**Methods:**

Phase 1 was piloted in an education district with 14 Primary 1 teachers assessing a cohort of 154 children, following which the instrument was adapted for the Scottish context (Scottish Early Development Instrument: SEDI). Phase 2 was then carried out using the SEDI. Data were analysed from a larger sample of 1090 participants, comprising all Primary 1 children within this school district, evaluated by 68 teachers.

**Results:**

The SEDI displayed adequate psychometric and discriminatory properties and is appropriate for use across Scotland without any further modifications. Children in the lowest socioeconomic status quintiles were 2–3 times more likely than children in the most affluent quintile to score low in at least one developmental domain. Even in the most affluent quintile though, 17% of children were ‘developmentally vulnerable’, suggesting that those in need cannot be identified by socioeconomic status alone.

**Conclusions:**

The SEDI offers a feasible means of providing communities with a holistic overview of school readiness for targeting early years’ interventions.

## Background

Evidence on the effectiveness of early childhood interventions is accumulating [1]. Early development has been shown to influence proximal factors such as behaviour, academic outcomes, and school drop-out rates [2], and later life outcomes such as social participation, criminality, obesity, and mental health [3,4]. Furthermore, neurodevelopmental research confirms that the brain is most malleable in the early years [5,6], hence both public health professionals and economists argue that investing in this period is necessary for tackling inequality [7,8]. Indeed monetary returns to society over an individual’s life span are expected to more than repay the initial investment [9]. Social gradients in health outcomes have been reported for infant and child mortality, low birth weight, injuries, malnutrition, infectious disease and usage of healthcare facilities [10]; and in cognitive outcomes for school admission, mathematical and language attainment, and literacy [11]. Public health professionals have consequently called for studies on interventions aimed at reducing socioeconomic inequalities in healthy child development [12].

Dubbed the ‘sick man of Europe’ [13], Scotland is a fitting location for addressing inequalities observed across a range of health outcomes [14-16]. Unfortunately should appropriate interventions be implemented, it would be difficult to determine their success on developmental and health outcomes as, with a few exceptions such as child dental health and weight at school entry, routine population-level measures currently collected in Scotland are mostly concerned with either birth, or later life end-stage events [17]. There is a strong case for more ‘upstream’ population outcome measures, taken earlier in the life-course, that predict later life chances and wellbeing outcomes and which may be changed within the usual policy-maker time horizon, say five years, by intervention [17,18]. Commonly used individual-level child development measures are typically designed to be administered by particular groups of education and health professionals such as psychologists, or physicians, thus making population-level data collection unfeasible or at least very costly. Moreover they focus on specific developmental domains such as language, cognitive or physical development, rather than providing a holistic measure. These measures, though useful for the purposes for which they were designed, are less useful as ‘upstream’, routinely-collected outcome measures suitable for community use.

### The Early Development Instrument

The Early Development Instrument (EDI) [19,20] was devised in Canada in response to this need for a universal, holistic, sensitive population measure of early child development outcomes that measures how well communities prepare their children for school [21]. It has been extensively validated [22,23], and is now used in Canada and Australia, and parts of the USA, Central and South America, Asia, the Middle East, Africa and Europe. It provides a holistic, community-level overview of child development with findings readily accessible and interpretable by health, educational and social work agencies for planning and evaluation purposes. Class teachers complete the EDI for all school entrants, typically children aged 4–6 years, based on their knowledge of the children’s skills, competences and behaviour after four months of classroom contact. The instrument evaluates five key developmental domains: *Physical health and wellbeing*, *Social competence*, *Emotional maturity*, *Language and cognitive development*, and *Communication and general knowledge*. The EDI is never interpreted or reported at the individual child level or used for clinical diagnostic purposes. Instead, it is intended for community-level monitoring and as a catalyst in planning and action to improve aspects of the local environment that influence early child development [19]. Results are fed back to communities, allowing local stakeholders, planners and policy makers to judge developmental progression in order to reflect on the effectiveness of support services, and to locally plan early childhood interventions for infants, children, parents, families, and communities that address identified collective developmental vulnerabilities and so better prepare children for school.

EDI items are scored from 0–10 for each developmental domain. A mean domain score is then calculated. Children whose scores fall in the bottom 10% are classed as vulnerable in that domain. If scores are beneath the 10th percentile on one or more domains, then children are classed as ‘overall developmentally vulnerable’ (Note that in EDI terminology, ‘vulnerable’ does not have connotations with child protection or social services but merely refers to children scoring within the lowest 10%). EDI results can then be reported in three ways. The first is mean scores (out of ten) for each domain. Secondly, the percentage of children who are ‘overall developmentally vulnerable’ can be reported. Finally, for each of the five domains of development, the percentage of children in an area scoring in the bottom 10% (‘vulnerable’), between 10 and 25% (‘at risk’), and above 25% regarded as ‘on track’ for development, are reported. This uses 10th and 25th percentile ‘cut-offs’ scored by the whole cohort. Each can be examined at different geographical levels: region, neighbourhood or school level. Indeed, it is common practice in both British Columbia (Canada) and Australia to depict local communities’ EDI results on coloured maps, to facilitate the use of these data by the general public [18,21].

In Canada, data linkage of routinely-collected population level measures of numeracy, reading comprehension and writing skills with the EDI dataset was conducted [24]. This allowed them to develop the Community Index of Child Development, a means of summarising development in individuals over time, rather than relying on cross-sectional data of different sets of children. Data linkage experts in Manitoba have also linked databases to explore the role of the socioeconomic gradient and infant health in child development, the comparative influence of family and neighbourhood in later wellbeing, and the long-term effects of poverty reduction [25,26].

### The Scottish context

In Scotland more than 95% of 4-year olds attend free pre-school education funded by local authorities [27]. Children start attending formal school at age 4.5 to 5.5 years, depending on birth dates, in August each year and only 1% of children attend an independent (non-government funded) school [27]. This first year of formal school is called Primary 1 (P1) and is broadly equivalent to the reception year in England and Wales, and the kindergarten year in Canada and Australia.

Within Scotland, however, there have been few means by which geographical or socioeconomic inequalities in child development have been widely and consistently measured to identify inequalities in early childhood. The aim of this study was to pilot a Scottish EDI within one school district in central Scotland. There were two phases of data collection with the following objectives:

Phase 1:

• to test the feasibility of implementing the Canadian EDI within the Scottish school context with a small sample

• to adapt the Canadian EDI to the Scottish context

Phase 2:

• to incorporate Phase 1 adaptations into a Scottish version (Scottish Early Development Instrument: SEDI) implemented across the whole school district

• to provide the community with information about children’s strengths and vulnerabilities

• to assess the psychometric properties of the SEDI (e.g. internal reliability of the questionnaire items) and its discriminatory ability, that is the tool’s ability to identify communities where children are well prepared for school compared to communities where children and their teachers are managing complex challenges

• to establish the tool’s feasibility for use with a wider Scottish population.

## Methods

### Measure

The EDI questionnaire evaluates five domains of child development: *Physical health and wellbeing* (items A2-A13 and C58/17–28 and 126); *Social competence* (items C1-C25 and C27, 69–93 and 95); *Emotional maturity* (items C28-C57/96–125); *Language and cognitive development* (items B8-B33/36–61); and *Communication and general knowledge* (items B1-B7 and C26/29-35 and 94). In addition, all domains, with the exception of *Communication and general knowledge*, comprise a number of sub-domains. For example, the *Physical health and wellbeing* domain comprises the sub-domains, *Gross and fine motor skills*, *Physical readiness for the school day* and *Physical independence*[20].

### Procedure

Both phases of this EDI pilot study were conducted in a Scottish local authority district where new initiatives to improve early years’ outcomes had recently been implemented, and thus much local support had been raised for early years’ interventions – including this pilot study. Paper-based questionnaires were used in both phases. Teachers completed the assessments in their normal working hours while substitute teachers, funded by the study, were provided to teach their classes. One hundred and fifty-four P1 pupils (82 female, 72 male) from the school district participated in Phase 1. Questionnaires were completed by 14 teachers. Mean pupil age was 5 years 7 months (SD = 4 months, range 4 years 3 months - 6 years 9 months). Qualitative methods, including focus groups and semi-structured questionnaires, were used to gather feedback from teacher participants on the tool and the process. Cronbach’s α was used to determine internal consistency. Phase 1 results suggested that the EDI could be feasible to administer and implement in Scotland and that only minor changes were required to reflect the Scottish context, e.g., changing ‘repeating grade’ to ‘repeating Primary 1’, changing teaching qualifications to denote recognised Scottish qualifications, and to reduce administration errors, e.g., pre-entering part of year details for child date of birth and date of completion.

The larger Phase 2 sample was recruited from all 1180 P1 pupils in the school district from its 35 schools, including independent schools. Sixty-eight P1 teachers completed the EDI questionnaires (100% teacher response rate). Eighteen children were opted-out^a^ of participation by their parents. An additional 72 children were excluded because they had been in school for less than one month, or had missing data, or had special needs^b^. Pupil postcodes were grouped into five quintile categories using the Scottish Index of Multiple Deprivation (SIMD)^c^, a postcode-based index of socioeconomic status assigned to each of 6,505 data zones across Scotland’s population of about 5 million, on the basis of census, unemployment and other data. A score of 1 was given to the most deprived area and 5 the most affluent. For seven children there was no quintile information available hence they were excluded from results requiring socioeconomic status quintile.

### Sample characteristics

The final sample then consisted of 1090 sets of pupil data (524 females, 563 males, 3 where gender was not recorded). Mean age was 5.51 years, SD = 0.32, range = 4.49 – 6.94. Most had English as their first language with 26 pupils identified as speaking English as well as another language. Seven pupils spoke only Polish or Urdu.

### Ethics

The research was approved by: the School of Psychological Sciences and Health Ethics Committee of the University of Strathclyde, Glasgow, UK; the Education Authority of the relevant school district; and the Chief Scientist Office of the Scottish Government. In line with EDI data collection in other countries, opt-out consent was utilized for parents of pupils. Parents received detailed information sheets with clear instructions on how to refuse participation of their children. All teachers provided written, informed consent.

## Results

### Reliability

Cronbach’s α for the SEDI overall was .97, indicating high levels of internal reliability. Cronbach’s α for four of the five individual domains was greater than .9, and the fifth was .78, showing good reliability. Sub-domains also indicated good levels of reliability except for *Physical independence* and *Physical readiness for school day* with Cronbach’s α of .22 and .51 respectively. Item deletion did not significantly improve reliabilities. Indeed, a reliability of .26 reported for a Canadian sample was similarly poor for the *Physical independence* sub-domain [20]. Five items were noted as having low item-total correlations (0.2). *Independent in toilet habits most of time (Physical independence sub-domain of Physical wellbeing* scale); *sucks thumb/finger, upset when left by parent/guardian* and *is shy (Emotional maturity*); and *knows how to handle a book* (*Language and cognitive*).

### Mean scores by domain

All *yes/no* scores and three-point Likert scores on the SEDI were converted into scores on ten-point scales to allow comparison across domains. Tables 1 and 2 summarize mean scores, score ranges and percentile cut-offs of score distributions, by EDI domain and gender.

**Table 1:** **School enterers mean score and 10/25/50**% **cut-off scores in overall sample by EDI domain**

**Domain**	**Score**			**Cut-off**		
	**Min**	**Max**	**Mean (SD)**	**10%**	**25%**	**50%**
Physical health and wellbeing (n = 13 items)	2.31	10.00	8.89 (1.30)	7.31	8.08	9.23
Social competence (n = 26 items)	0.00	10.00	8.64 (1.71)	5.96	7.88	9.42
Emotional maturity (n = 30 items)	2.41	10.00	8.31 (1.32)	6.50	7.67	8.57
Language and cognitive development (n = 26 items)	0.38	10.00	8.89 (1.59)	6.92	8.46	9.62
Communication and general knowledge (n = 8 items)	0.00	10.00	8.39 (2.25)	5.00	6.88	10.00

**Table 2:** EDI developmental vulnerability of school enterers by gender and domain

**Gender & domain**	**Mean score out of 10 (SD)**	**Developmentally vulnerable**	**Developmentally at risk**	**On track**	
		**In the lowest 10%**	**Between the 10th and the 25th percentile**	**Between the 25th and 50th percentile**	**Above the 50% percentile**
		**%**	**%**	**%**	**%**
Male					
PHWB	8.71 (1.39)	18.7	14.1	26.0	41.3
SC	8.36 (1.79)	13.0	18.2	30.0	38.8
EM	7.98 (1.37)	15.4	22.2	22.8	39.6
L&C	8.67 (1.74)	13.4	12.2	46.1	28.3
C&GK	8.02 (2.35)	15.5	17.4	67.1	0
Female					
PHWB	9.08 (1.16)	10.3	12.8	26.0	51.0
SC	8.94 (1.57)	7.9	10.0	22.5	59.6
EM	8.67 (1.17)	6.1	12.3	20.3	61.3
L&C	9.14 (1.36)	7.4	9.4	45.0	38.2
C&GK	8.78 (2.06)	8.6	10.9	80.5	0

PHWB = Physical Health and Wellbeing; SC = Social Competence; EM = Emotional Maturity; L&C = Language and Cognition; C&GK = Communication and General Knowledge.

### SEDI vulnerability by domain and by SES quintile

**Figure 1:**
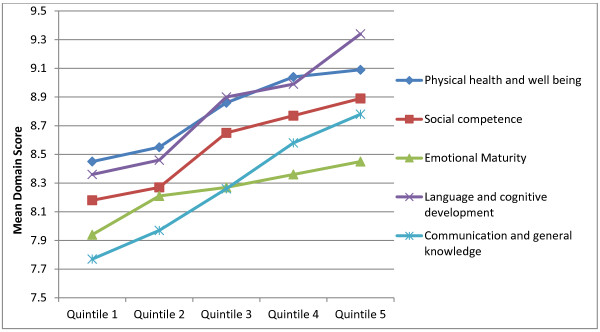
**EDI mean domain score of school enterers by socioeconomic status quintile.** The Scottish Index of Multiple Deprivation (SIMD) ranks small postcode areas (called data zones) according to level of deprivation by combining indicators such as current income, employment, health, education, skills, housing, and crime. Data zones can then be grouped into quintiles (five groups). Quintile 5 is most affluent and quintile 1 least affluent.

Scores were then further categorised according to SES quintile groups (Figure 1). For each SEDI domain, quintile scores appeared to follow a SES gradient. One-way analysis of variance indicated significant differences between quintile groups for *Physical health and wellbeing* (*F* (4, 1081) = 7.55, *p* = .000); *Social competence* (*F* (4, 1082) = 5.07, *p* = .000); *Language and cognitive development* (*F* (4, 1082) = 9.69, *p* = .000) and *Communication & general knowledge* (*F* (4, 1082) = 5.11, *p* = 0.000). No significant differences between quintiles were found in the *Emotional maturity* domain (F (4, 1079) = 1.78, p > .05). Allowing that there was only a small quintile 1 group (4%) in this sample, results suggested that the SEDI can discriminate school readiness skills across socioeconomic gradients.

### Domain cut-offs: vulnerable, at-risk and on-track by quintile

Mean scores for the sample and 10%, 25% and 50% cut-off vulnerability scores for each domain were used to calculate the frequency and percentage of children categorised as developmentally vulnerable in each quintile for each SEDI domain, i.e., those with scores below the 10th percentile. In addition, children whose score fell between the 10th and the 25th percentile were classed as ‘at-risk’, those whose scores were between the 25th percentile and 50th percentile were ‘on-track 1’, and those above the 50th percentile were ‘on-track 2’. Tables 2 and 3 show vulnerability frequencies and percentages for each domain, by gender and by SES quintile group.

**Table 3:** EDI developmental vulnerability of school enterers by SIMD quintile and domain

**SIMD quintile & domain**	**Mean score out of 10 & (SD)**	**Developmentally vulnerable**	**Developmentally at risk**	**On track**	
		**In the lowest 10%**	**Between the 10th and the 25th percentile**	**Between the 25th and 50th percentile**	**Above the 50% percentile**
		**%**	**%**	**%**	**%**
1: Most deprived					
PHWB	8.45 (1.67)	25.6	12.8	28.2	33.3
SC	8.18 (1.97)	20.6	8.8	35.3	35.3
EM	7.94 (1.52)	15.8	21.1	28.9	34.2
L&CD	8.36 (1.70)	18.9	18.9	45.9	16.2
C&GK	7.77 (2.66)	17.9	17.9	64.1	0
2: Deprived					
PHWB	8.55 (1.38)	22.4	18.1	25.4	34.1
SC	8.27 (1.91)	14.4	19.1	29.8	36.7
EM	8.21 (1.44)	13.9	16.5	25.1	44.6
L&C	8.46 (1.93)	19.0	11.1	43.5	26.4
C&GK	7.97 (2.47)	18.1	16.8	65.1	0
3: Average					
PHWB	8.86 (1.31)	14.6	14.6	26.5	44.2
SC	8.65 (1.73)	11.9	12.4	23.3	52.4
EM	8.27 (1.36)	11.9	19.0	17.3	51.8
L&C	8.90 (1.61)	10.5	14.3	34.3	41.0
C&GK	8.26 (2.33)	15.9	14.2	69.9	0
4: Affluent					
PHWB	9.04 (1.28)	12.8	8.6	25.6	53.0
SC	8.77 (1.63)	8.6	12.6	25.7	53.1
EM	8.36 (1.26)	9.7	18.1	20.8	51.4
L&C	8.99 (1.51)	8.8	8.3	53.5	29.5
C&GK	8.58 (2.11)	9.4	12.1	78.6	0
5: Most affluent					
PHWB	9.09 (1.05)	6.7	17.9	25.1	50.3
SC	8.89 (1.42)	6.3	15.0	26.3	52.5
EM	8.45 (1.17)	7.3	14.6	23.0	55.1
L&C	9.34 (0.93)	1.8	10.7	42.6	45.0
C&GK	8.78 (1.93)	5.6	15.0	79.4	0

SIMD = Scottish Index of Multiple Deprivation.

Quintile 1: N = 39, Quintile 2: N = 232, Quintile 3: N = 226, Quintile 4: N = 406, Quintile 5: N = 180.

PHWB = Physical Health and Wellbeing; SC = Social Competence; EM = Emotional Maturity; L&C = Language and Cognition; C&GK = Communication and General Knowledge.

Inspection of these figures demonstrates that the majority of participants were ‘on-track’, regardless of quintile group or domain. The *Communication and general knowledge* domain further displayed ceiling effects for all quintile groups highlighting that for this SEDI component children from all quintiles tended to score highly, although this part of Scotland does not contain the most deprived areas.

### Developmental vulnerabilities in one, two or more domains by quintile

**Figure 2:**
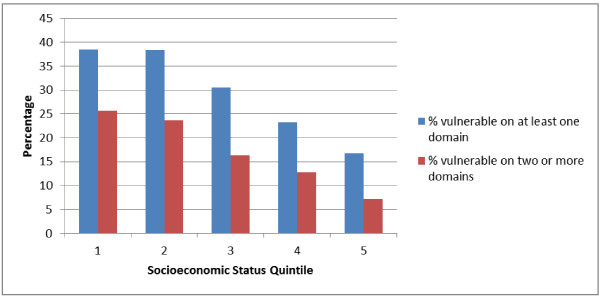
**Percentage children vulnerable on one or more, and two or more developmental domains by quintile.** Vulnerable=Scoring in the bottom 10% in a developmental domain. The five developmental domains were: Physical health and wellbeing; Social competence; Emotional maturity; Language and cognitive development; and Communication and general knowledge. Quintile 5 is most affluent and quintile 1 least affluent.

Data were then examined for developmental vulnerability in one, and two or more, domains. 297 children in the sample (27.3%) were recorded as vulnerable in one or more domains, and 168 (15.4%) in two or more domains. Developmental vulnerabilities by SIMD quintile can be seen in Figure 2. Quintile 5, the most affluent SES grouping, showed the lowest vulnerability rates (16.7%) in one or more domains, and quintile 1, the least affluent, demonstrated the highest rate of vulnerability (38.5%). Figure 2 also illustrates percentage vulnerabilities in two or more domains by quintile. A similar pattern of results was found, with quintile 5 (the most affluent areas) showing the lowest vulnerability rates (7.2%) and quintile 1 the highest vulnerability rate (25.6%).

Analysis of our results by gender showed that 193 boys (34.3% boys) and 104 girls (19.8% girls) were vulnerable in one domain. Adjusted for age, the odds ratio of a boy being vulnerable in one domain was 2.38 times that of a girl. Similarly 111 boys (19.7%) and 56 girls (10.7%) were vulnerable in two or more developmental domains. Adjusted for age, the odds of a boy being vulnerable in at least two domains were 2.51 times that of a girl. The odds of a child who did not communicate adequately in his/her first language presenting as vulnerable in two or more domains were 8.04 times that of children who were able to communicate adequately in their first language.

Analysis by SIMD quintile, adjusted for age and gender, showed that in the most deprived quintile (1) children were 2.77 times more likely than those in the most affluent quintile (5) to be vulnerable in one or more domains. Compared to children in quintile 5, children in quintile 2 were 3.04 times more likely, quintile 3 were 2.21 times more likely, and quintile 4 were 1.45 times more likely, to be vulnerable in one or more domains. Similarly for vulnerability in two or more domains, children in quintile 1 were 3.36 times more likely to be vulnerable than those in quintile 5. Children in quintile 2 were 3.68 times more likely, quintile 3 were 2.09 times more likely and quintile 4 were 1.61 times more likely, to be vulnerable in at least two domains compared to children in quintile 5.

Chi square analyses also showed each of the following indicators to be significantly associated with vulnerability on one or more domains: child struggling to do his/her school work; child needing further assessment; child on a waiting list for further assessment; and child receiving school based supports.

### Cost

Finally, we costed the data collection process for the EDI in the school district, based on actual Scottish costs of teacher time buy-out, i.e. to purchase substitute teacher services, for training (one half day) and EDI completion (about one day). If we assume direct web-based data-entry by teachers, as is normal in Canada and Australia, then the total cost of data collection and entry comes to about £20 sterling per child, 95% of which goes to teacher time buy-out. This would translate, for an “average” Scottish Local Authority with a total population of 150,000 and a crude birth rate of about 1%, to £30,000 for each wave of data collection – typically every three calendar years in Canada and Australia. Thus, for the average Local Authority the annualized cost would be £10,000.

## Discussion

The study’s aim was to test the usefulness of a Scottish version of the EDI as a tool to assess global development in Scottish children at school entry. Results showed that it displayed adequate psychometric and discriminatory properties. Internal reliabilities for domains were good, ranging from .78 to .96. Five items identified as having low item-total correlations (*independent in toilet habits most of time, sucks thumb/finger, upset when left by parent/guardian’, is shy* and *‘knows how to handle a book’*) should be monitored in any further Scottish roll-out. Mean domain scores demonstrated the expected social gradient across five SES postcode-based quintiles: Quintile 5, the most affluent quintile, had the highest mean scores across all domains and quintile 1 the lowest.

As well as providing information across a region, EDI results can contribute to international comparisons. For example, mean domain scores for our Scottish school district sample were slightly higher on all five domains than those reported for Canadian children [28] and slightly lower than reported Australian scores [29]. Nonetheless our school district scores followed the same pattern as the Australian and Canadian studies, suggesting that English-speaking Western children may have similar shortfalls and strengths in their early development. Percentage vulnerability in one or more domains for our sample (27.3%) was also similar to those reported in EDI studies in Canada (27.2%) and Australia (23.5%) [28,29]. Gender differences in the Scottish sample were also similar to those reported in other countries where boys were more likely to be developmentally vulnerable than girls [30]. However vulnerability in two or more domains was 15.4% for our Scottish district sample, which appears higher than 12.4% reported for the Canadian sample, and 11.8% for Australia [28,29]. At this point we do not know whether this local data represents developmental vulnerabilities across the whole of Scotland. Nonetheless it exemplifies the kind of information that EDI results produce for policy makers’ consideration.

Although an SES gradient of vulnerability by quintile is evident, the study findings also highlighted that significant developmental vulnerability was found across all five quintiles and not only in the most deprived. Such detailed data provide evidence needed to implement what Michael Marmot has called “proportionate universalism,” [8] including targeting of local programmatic interventions tailored to individual developmental domains and delivered in those communities where it is most needed. This is particularly relevant in Scotland where, in 2005, the core Child Health Surveillance programme was cut back in that the number of routine health visitor contacts in the first four years of life was reduced by three contacts thereby limiting opportunities to identify children and families with problems [31,32]. In addition, the government utilizes a decentralised model allowing local authorities and/or health boards to make their own decisions with regard to additional services. Thus different parts of the country are using different licensed parenting programmes such as Triple P (Glasgow Health Board), Incredible Years (West Lothian Council), Mellow Parenting and numerous others [1]. Some programmes, such as David Olds’ Family Nurse Partnership, have been centrally funded and managed, starting with a ‘test site’ in NHS Lothian, Edinburgh, and subsequently being rolled out to limited sites in NHS Tayside, Greater Glasgow and Clyde, Ayrshire and Arran, Fife, Lanarkshire and Highland. Wider adoption of the EDI, or indeed any single measure of early child development, is currently therefore difficult to achieve in Scotland, due to decentralization, and the attendant local heterogeneity in stakeholder practices. However, adopting the EDI may provide stakeholders with knowledge about their communities enabling them to make wise choices with regard to child and parenting programmes.

In other regions that have utilised EDI evaluation, it has acted as a catalyst for public health, social work and education to collaborate to examine available resources for families with young children and identify areas of additional need to improve young children’s opportunities for achievement [21]. Furthermore, there is suggestive evidence from early Western Australian use of the EDI, in disadvantaged communities such as Mirrabooka, that community EDI scores can be substantially shifted within a half-decade, using improved local preschool programming [33]. This contrasts with epidemiological conventional wisdom regarding the much longer lag-times required to change the usual, routinely collected health indicators (mostly based on birth, hospitalization and death) which typically require much longer time-horizons for their alteration, including the reduction of health inequalities by social class [17].

The SEDI uses teachers’ ratings of aspects of children’s development hence reporting bias is a potential limitation of the study. A vast body of literature exists which considers proxy versus direct assessment of child development and wellbeing. Reviews of child wellbeing assessment using Health-Related Quality-of-Life (HR-QOL) instruments considered parent-provided and self-rated approaches. In several studies a high correlation was shown for ‘observable’ components such as physical, and a low correlation for non-directly observable components such as emotional or social areas [34,35]. Frameworks have been developed to delineate between various proxy perspectives and to guide inquiries into the validity and interpretation of viewpoints [36]. The age of children has been found to have a moderating function on the closeness of correspondence [35]. In fact, 5-year-old children have been found unable to understand a sufficient number of items to describe their health adequately [37].

The EDI assesses physical, cognitive, emotional and social aspects of children 4–6 years. Reliability testing has been conducted by the designers of the EDI [38-40]. Both test-retest reliability (using the Pearson correlation coefficient) and intra-rater or within-teacher reliability (using the intra-class correlation coefficient) tests have been conducted. Both have shown high coefficients of between 0.7 and 0.96 [38].

## Conclusion

The SEDI’s psychometric properties are shown to be robust, and it is able to highlight developmental differences in children between socioeconomic and geographic areas. The tool’s simplicity and usability lend themselves easily to community-wide implementation across Scotland without further modification. It has the potential to offer communities a holistic overview of school readiness in their children, to both fuel support and provide a baseline for targeted interventions.

## Endnotes

^a^The opt-out group comprised 11 females and 7 males. One of the opt-out pupils had been in class less than one month; two pupils’ data were recorded ‘opt-out’ because they had moved school.

^b^Of the further 72 pupils excluded, 42 were recorded as having special needs and 30 pupils had been in class for less than one month or the length of time in class had not been recorded. Participants who had 30% of the questionnaire items missing and all those who had more than 1 scale of domain data missing were excluded. Only 1 pupil fell into the latter category and had already been excluded as he/she had been in the class for less than one month.

^c^The Scottish Index of Multiple Deprivation (SIMD) ranks small postcode areas (called data zones) according to level of deprivation by combining indicators such as current income, employment, health, education, skills, housing, and crime. Data zones can then be grouped into quintiles (five groups).

## Competing interests

The authors declare that they have no competing interests.

## Authors’ contributions

RG and JF conceived and designed the study. RG obtained, prepared and managed the data. SM and JNB performed the statistical analysis under the supervision of LMW. LMW, SM, JNB, RG and JF interpreted the findings. LMW, JF and RG drafted the manuscript. All authors read and approved the final manuscript.

## Pre-publication history

The pre-publication history for this paper can be accessed here:

http://www.biomedcentral.com/1471-2458/13/1187/prepub

## References

[B1] Geddes R Haw S Frank J Interventions for Promoting Early Child Development for Health: An Environmental Scan with Special Reference to Scotland 2010 Edinburgh: Scottish Collaboration for Public Health Research and Policy

[B2] Commission on Social Determinants of Health Closing the gap in a generation: health equity through action on the social determinants of health Final Report of the Commission on Social Determinants of Health 2008 Geneva: WHO

[B3] Irwin L Siddiqi A Hertzman C Early child development: A powerful equalizer. Final Report for the WHO Commission on the Social Determinants of Health 2007 Geneva: WHO

[B4] Kuh D, Ben-Shlomo Y A life course approach to chronic disease epidemiology 2004 Oxford: Oxford University Press

[B5] McCain M Mustard J Reversing the real brain drain: Early years study Final report 1999 Toronto: Publications Ontario

[B6] Shonkoff J Phillips D From neurons to neighbourhoods: The science of early childhood development 2000 Washington, DC: National Academy Press 25077268

[B7] Heckman J Skill formation and the economics of investing in disadvantaged children Science 2006 13 1900 1902 10.1126/science.1128898 16809525

[B8] Marmot M Fair society, healthy lives 2010 London: University College London

[B9] Ludwig J Phillips D The benefits and costs of head start Social Policy Report 2007 13 3 19

[B10] Hertzman C Tackling inequality: get them while they’re young BMJ 2010 13 346 348 10.1136/bmj.c346

[B11] Smith J Brooks-Gunn J Klebanov P Duncan GJ, Brooks-Gunn J Consequences of living in poverty for young children’s cognitive and verbal ability and early school achievement Consequences of growing up poor 1997 New York: Russell Sage 132 189

[B12] Dunn JR Socio-economic inequalities in healthy child development: The evidence grows J Epidemiol Community Health 2012 13 193 10.1136/jech-2012-201040 22308297

[B13] McCartney G Walsh D Whyte B Has Scotland always been the ‘sick man’ of Europe? An observational study from 1855 to 2006 Eur J Public Health 2012 13 756 760 10.1093/eurpub/ckr136 22021374 PMC3505444

[B14] Gray R Bonellie S Chalmers J Contribution of smoking during pregnancy to inequalities in stillbirth and infant death in Scotland 1994–2003: retrospective population based study using hospital maternity records BMJ 2009 13 b3754 10.1136/bmj.b3754 19797343 PMC2755727

[B15] Levin K Davies C Topping G Inequalities in dental caries of 5-year-old children in Scotland, 1993–2003 Eur J Public Health 2009 13 337 342 10.1093/eurpub/ckp035 19307245

[B16] Scottish Government Health Analytical Services Division Long-term monitoring of health inequalities First report on headline indicators 2008 Edinburgh: Scottish Governmen

[B17] Frank J Haw S Best practice guidelines for monitoring socioeconomic inequalities in health status: lessons from Scotland Milbank Q 2011 13 658 693 10.1111/j.1468-0009.2011.00646.x 22188351 PMC3250637

[B18] Hertzman C Williams R Making early childhood count CMAJ 2009 13 68 71 10.1503/cmaj.080512 19124792 PMC2612056

[B19] Janus M Offord D Developmental and psychometric properties of the early development instrument: a measure of children’s school readiness Can J Behav Sci 2007 13 1 22

[B20] Janus M Walsh C Duku E Early Development Instrument: Factor structure, sub-domains and multiple challenge index 2005 Hamilton: Offord Centre for Child Studies, McMaster University

[B21] Goldfield S Sayers M Brinkman S The process and policy challenges of adapting and implementing the early development instrument in Australia Early Educ Dev 2011 13 911 978

[B22] Forer B Zumbo B Validity of multilevel constructs: Validation findings and empirical methods for the EDI Soc Indic Res 2011 13 231 235 10.1007/s11205-011-9844-3

[B23] Janus M Brinkman S Duku E Validation and psychometric properties of the early development instrument in Canada, Australia, United States and Jamaica Soc Indic Res 2011 13 283 297 10.1007/s11205-011-9846-1

[B24] Lloyd J Hertzman C From kindergarten readiness to fourth-grade assessment: longitudinal analysis with linked population data Soc Sci Med 2009 13 111 123 10.1016/j.socscimed.2008.09.063 18986743

[B25] Roos L Brownell M Lix L From health research to social research: privacy, methods, approaches Soc Sci Med 2008 13 117 129 10.1016/j.socscimed.2007.08.017 17919795

[B26] Roos N Roos L Brownell M Enhancing policymakers’ understanding of disparities: relevant data from an information-rich environment Milbank Q 2010 13 382 403 10.1111/j.1468-0009.2010.00604.x 20860576 PMC3000932

[B27] Scottish Government Growth and development – Pre-school education 2013 Edinburgh: Scottish Government Available at http://www.scotland.gov.uk/Topics/Statistics/Browse/Children/TrendNursery (accessed 16 September 2013)

[B28] Offord Centre for Child Studies Updated normative II Offord Centre for Child Studies, McMaster University, Canada, 2012 Available at http://www.offordcentre.com/readiness/reports.html (accessed 13 November 2012)

[B29] Centre for Community Child Health and Telethon Institute for Child Health Research A snapshot of early childhood development in Australia – AEDI National Report 2009 Canberra: Australian Government Department of Education

[B30] Janus M Duku E Result of the Phase II implementation of the EDI in Scotland. Technical Report 2012 Hamilton: Offord Centre for Child Studies, McMaster University

[B31] Wright CM Jeffrey SK Ross MK Wallis L Wood R Targeting health visitor care: lessons from starting well Arch Dis Child 2009 13 23 27 10.1136/adc.2007.136465 18456687

[B32] Wood R Wilson P General practitioner provision of preventive child health care: analysis of routine consultation data BMC Fam Pract 2012 13 73 10.1186/1471-2296-13-73 22862924 PMC3460766

[B33] Royal Children’s Hospital Centre for Community Child Health in Melbourne, the Murdoch Children’s Research Institute, and the Telethon Institute for Child Health Research Mirrabooka Community, Western Australia 2010 Melbourne: Royal Children’s Hospital Available at http://training.aedi.org.au/Secondary-Pages/About-the-video-case-studies/Mirrabooka-Community-Western-Australia.aspx (accessed 18 January 2013)

[B34] Ravens-Sieberer U Erhart M Wille N Wetzel R Nickel J Bullinger M Generic health-related quality-of-life assessment in children and adolescents: methodological considerations Pharmacoeconomics 2006 13 12 1199 1220 10.2165/00019053-200624120-00005 17129075

[B35] Eiser C Morse R Can parents rate their child’s health-related quality of life? Results of a systematic review Qual Life Res 2001 13 4 347 357 10.1023/A:1012253723272 11763247

[B36] Pickard AS Knight SJ Proxy evaluation of health-related quality of life: a conceptual framework for understanding multiple proxy perspectives Med Care 2005 13 5 493 499 10.1097/01.mlr.0000160419.27642.a8 15838415 PMC1188232

[B37] Rebok G Riley A Forrest C Starfield B Green B Robertson J Tambor E Elementary school-aged children’s reports of their health: a cognitive interviewing study Qual Life Res 2001 13 1 59 70 10.1023/A:1016693417166 11508476

[B38] Duku E Janus M Stability and reliability of the Early Development Instrument: A population based measure for communities (EDI) 2004 Hamilton, Ontario, Canada: Paper presented at the 16th Annual Research Day, Department of Psychiatry and Behavioural Neurosciences, McMaster University

[B39] Janus M Offord D Development and psychometric properties of the early development instrument (EDI): a measure of children’s school readiness Can J of Beh Sc 2007 13 1 1 22

[B40] Janus M Hertzman C Guhn M Brinkman S Goldfeld S Reply to Li, D’Angiulli and Kendall: the early development index and children from culturally and linguistically diverse backgrounds Early Years: An International Journal of Research and Development 2009 13 1 83 87 10.1080/09575140802689125

